# Quitters referring smokers: a quitline chain-referral pilot study

**DOI:** 10.1186/1756-0500-7-282

**Published:** 2014-05-05

**Authors:** Kathryn L DeLaughter, Julie E Volkman, Barrett D Phillips, Thomas K Houston

**Affiliations:** 1 eHealth Quality Enhancement Research Initiative, Bedford, VA, USA; 2 University of Massachusetts Medical School, Worcester, MA, USA

**Keywords:** Quitline, Smoking cessation, Social networking

## Abstract

**Background:**

Telephone counseling Quitlines can support smoking cessation, but are under-utilized. We explored the use of smoker peer-referrals to increase use of a Quitline in Mississippi and Alabama.

**Findings:**

Collaborating with the Alabama and Mississippi Quitline, we piloted peer-referrals to Quitlines. Successful ‘quitters’ who had used the Quitline were contacted at routine follow-up and recruited to participate as a peer-referrer and refer their friends and family who smoked to the Quitline. Peer-referrers completed a training session, received a manual and a set of Quitline brochures a peer-referral forms. These peer-referral forms were then returned to the Quitline telephone counselors who proactively called the referred smokers. Of the initial potential pool of 96 who quit using the Quitline, 24 peer-referrers (75% Women, 29% African-American, and high school graduates/GED 67%) were recruited and initially agreed to participate as peer-referrers. Eleven of the 24 who initially agreed were trained, and of these 11, 4 (4%) actively referred 23 friends and family over 2 months. From these 23 new referrals, three intakes (100% Women, 66% African-American) were completed. Of the initial pool of 96, 4 (4%) actively participated in referring friends and family. Quitline staff and peer-referrers noted several barriers including: time-point in which potential peer-referrers were asked to participate, an ‘overwhelming’ referral form to use and limited ways to refer.

**Conclusions:**

Though ‘quitters’ were willing to agree to peer-refer, we received a minority of referrals. However, we identified several areas to improve this new method for increasing awareness and access to support systems like the Quitline for smokers who want to quit.

## Findings

### Introduction

The most recent decade has witnessed a plateau effect with rates of smoking cessation holding steady, despite increasing effective treatments [1]. Smokers are disproportionately represented in harder-to-reach populations including lower-income and rural. Over sixty-percent of low-income smokers are interested in quitting, [2] but lack information about and access to smoking cessation resources [3,4] and too few try to quit each year [5,6]. In rural areas, access to clinical treatment is especially difficult [7,8]. New and innovative approaches are needed to access to cessation support for these smokers.

One solution is state-supported Quitlines, telephone counseling services smokers can call where lay coaches provide support and advice about quitting smoking. Nationally, Quitlines can be accessed toll-free (1-800-Quit-Now). Quitlines use short motivational interviews, focus on self-regulation, framing of decisions, and also promote the use of pharmaceutical aids in cessation attempts [9]. Despite evidence that Quitlines can increase cessation [10], they are still under-utilized (accessed by only 2% of smokers per year) [3,4]. Proactive referrals to Quitlines from clinical practices, fax-to-quit programs, have had success in increasing engagement by smokers, [11-14] but these programs are less available in rural, underserved areas [15].

In these hard-to-reach populations, peer navigators have been used successfully to engage patients in health promotion and disease management. Peer-led interventions have been effective in delivering culturally tailored behavioral interventions [16,17]. “Natural helpers” from the community trained to deliver health information can act as peer-navigators to increase health care access [18,19].

Peer navigation has not been evaluated in the context of connecting smokers to Quitlines. We assessed the feasibility of recruiting smokers who had successfully quit with support from a state-supported Quitline as peer-navigators, and examined the number of smokers who referred and completed intake into the Quitline services.

## Methods

### Study design

ProACT-2-Quit (“Proactive Access, Connectedness, and Telephone Counseling to Quit Smoking”) was a one-year, community-based, multi-element, Quitline-facilitated peer-navigation intervention integrated in the social network of low-income smokers. To address the multiple barriers in both reach of Quitline-delivered interventions for vulnerable groups, we designed a multi-element facilitation program to increase use of Quitline services. The Proact-2-Quit evaluation was approved by the University of Massachusetts Medical School Institutional Review Board.

The elements of the facilitation program drew from the constructs within Self-Determination Theory (relatedness, competence, and autonomy-support) [20] Peer-navigators who quit with support of the Quitline were from the same community and cultural background as those they were referring, enhancing relevance and relatedness of quitting and Quitline use. Peer-navigators were trained to tell their success story of quitting, acting as role models and enhancing the perception of competence in quitting. Peer-navigators were encouraged to elicit smokers’ own preferences for quitting, supporting the autonomy of the individual.

### Setting and sample

#### ***Mississippi and Alabama quitline***

Our partnering Quitline serves a large rural area which includes low-income, high-minority communities. The Alabama and Mississippi Quitline is administered through a state contract to Information and Quality Healthcare (IQH, http://www.iqh.org), the quality improvement organization of Mississippi. Our pilot represents a greater than national average percent of African Americans, as 26.5% of the population in Alabama and 37.3% population in Mississippi are African American, (13.1% of the nation reports being African American [21]). The percent of persons reporting below poverty level median is 17.6% in Alabama, with 21.6% in Mississippi, both considerably higher than the national median of 14.3% [21].

#### ***Standard quitline treatment***

Current standard Quitline treatment includes four counseling sessions, a “quit pack” of materials and information which includes free nicotine replacement therapy for up to 4 weeks.

### ProACT-2-Quit intervention

#### ***Recruiting and training peer-navigators***

The ProACT-2-Quit Peer-Navigators had access to more recently created chain-referral survey methods, including the more mathematically complex forms of respondent-driven sampling, which have quickly become the method of choice for recruiting hard-to-reach persons and their social networks, and have been adapted for use as a channel for peer-driven intervention delivery [22]. Chain-referrals are functionally “grassroots” and participatory; in line with the social network dynamics, and allows peer-driven access to high-risk groups within relatively short periods of time using a small number of initial Peer-Navigators. For four months in 2010, during the standard six-month follow-up call by the Quitline, ex-smokers (who had successfully quit 6 months prior) were recruited as peer-navigators.

Quitline staff then completed a telephone training session which lasted approximately 25 minutes, with the peer-navigators. They were trained to 1) briefly market the Quitline using their personal story of success, 2) persuade other smokers to sign a referral form and provide their telephone number, and 3) mail the peer referral form to the Quitline (Peer-navigators were sent 30 duplicate copies of the referral form, once completed, they were asked to return the bottom half in a postage-paid envelope and could mail as few or as many at a time, depending on how quickly they were completed (Additional file 1). Following the training session, Peer-Navigators were mailed all materials including an instruction sheet on how to refer their family and friends successfully, and a $50 honorarium.

#### ***Contacting smokers referred by peer navigators***

Using a protocol similar to that of a fax-referral, once Quitline staff received referral forms from the Peer-navigator, they proactively called the referred smoker to attempt to engage them in services.

### Data collection and analyses

We monitored the success of the chain referral process. We ran frequency analyses on the characteristics of our peer-referrers and those referred who completed the intake at the Mississippi Quit-Line. Finally, we analyzed qualitative feedback and field notes reported by the Quitline counselors.

## Results

### Recruiting peer-navigators

#### ***Sources of peer-navigators – active users of quitline services***

In the year prior to initiating the peer-referral program, the Mississippi and Alabama Quitline received 6,239 inquiries (any calls to the Quitline). Of these, 2,522 agreed to enter Quitline counseling, and 69% completed all four counseling sessions (1,735/2,522). Smokers were referred from many sources, including fax-to-quit referrals from health care providers (21.7%), television ads (27%) and radio ads (2%). Only 5.4% were reported as “word of mouth” referrals suggesting that in addition to general referral sources, the realm of peer-referrals need significant improvements.

#### ***Characteristics of current quitline-treated smokers***

Of the 2,522 people using Alabama Quitline counseling a year prior to the Peer-Referrals, the mean age was 44 (SD 13), 32% were female, and 24% were African-American. Although the Quitline does not currently collect income data, they do collect education and insurance. Of note, 53.6% (n = 1,433) had Medicaid or no insurance; only 10% of Quitline callers had a college degree, and 35% did not have a high school diploma.

As part of the state contract, the Quitline follows smokers to assess cessation rates. Based on Quitline data, of those who completed all four counseling sessions 19% (284/1557) completed 6-months telephone follow-up and reported quitting. These 284 smokers, the successful ones, were our target population. On average, 24 six-month follow-up with successful quitters are completed each month. Our Peer-Navigator recruitment was ongoing for four months, thus we recruited from a potential pool of 96 six-month successful quitters.

#### ***Success in recruiting peer navigators***

Between July and September 2010, 24 (25% of the potential pool of six-month successful quitters) ex-smokers agreed to be sent the information and participate in the study as a peer-navigator. Eleven (11% of potential pool) of these returned the consent form and participated in the training call with the Quitline staff in order to receive instruction on participation. The trained peer-referrers were mostly female (75%), and 29% were African-American (Table 1) and reflected the lower-educated population from which they were drawn.

**Table 1:** Characteristics of peer navigators

**Item**	**Peer navigator**		
	**Cohort (n)**	**Active (n)**	**Percent of cohort**
TOTAL	24	4	16.7%
**Sex (n)**			
Male	6	1	16.7%
Female	18	3	16.7%
**Age (n)**			
19-24	1	0	0%
25-34	5	1	20%
35-44	5	0	0%
45-54	7	2	28.6%
55-64	4	0	0%
65+	2	1	50%
**Highest grade of school (n)**			
College 1–3 yr	6	1	16.7%
College 4+ yr	-	-	-
Grade 12 or GED	16	3	18.8%
Grade 9-11	2	0	0%
**Ethnicity (n)**			
African American	7	2	28.6%
White	17	2	11.8%
**Previous cigarettes per day (n)**			
1-9	3	1	33.3%
10-19	6	1	16.7%
20-29	6	0	0%
30-39	5	1	20%
40-49	3	1	33.3%
50+	1	0	0%
**Does your spouse smoke/use tobacco? (n)**	Yes = 8	Yes = 0	0%
	No = 16	No = 4	25%

### Peer navigators referring new smokers

Of the eleven enrolled Peer-Referrers, four (4% from original potential pool) returned to the Quitline referral slips of the friends and family smokers they referred. These referral slips included a total of 23 smokers which represents 19% of the 120 referral slips provided to these four peer-referrers.

Counselors attempted to contact each of the referred smokers which subsequently resulted in 3 complete intakes. Of the 3 smokers who completed the intake, all were female and greater than 45 years old. One had completed some college; the other two had a high school diploma/GED. Two of the recruits were African American, one White. Two smoked 1 to 9 cigarettes per day and one recruit smoked more than 50 cigarettes per day.

### Field note review: identifying lessons learned

Counselors who dealt directly with peer-referrers identified several lessons learned. With the limited notes recorded by the Quitline staff, one theme noted by counselors was the time-point in which we contacted successful quitters to become peer-referrers. They felt that recruiting smokers at the beginning of quitting with counselor help (actively quitting smokers) would be better than waiting until six-month cessation. Next, the referral forms (Additional file 1) were considered a bit “overwhelming” to the peer-referrers. For future consideration, counselors noted that a large number of their smokers reported having text-enabled phones, and proposed a text-messaging based referral process might be more efficient and allow direct access to new smoker’s phone numbers.

## Discussion

From this low-income, hard-to-reach population, this pilot study demonstrated the challenges of peer-referrals. We were able to train 11 successful quitters to peer-refer those in their social network. However, only 3 peer-referred smokers were enrolled in the Quitline. Below, we provide additional detail on a number of barriers to using natural social networks to recruit smokers from rural populations, and implications for future research.

The successful quitters trained to be Peer-referrers generally reflected the population of smokers enrolled in the Quitline, but were more frequently women. Women and men approach smoking cessation differently, [23] with interventions being tailored to the circumstances surrounding quitting, reducing stigma and harm, obtaining social support and integration of social issues being most beneficial for women [24]. The opportunity to share their success story and address these issues may have contributed to a willingness to be a peer-referrer.

Overall, the ratio of intakes to referrals for this pilot project was approximately 0.13. In contrast, the ratio of intake to referrals is 0.32 fax-to-quit referrals from medical practices, as reported by the Quitline. As the Quitline number was on the peer-referral form, some additional smokers may have taken forms and self-referred instead of having their Peer-Navigators smoker send the form back to the Quitline. However, we cannot confirm these additional referrals.

One of the solutions recommended by the counselors at the Quitline was to recruit participants earlier, perhaps at the 4-week or 3-month follow-up, as opposed to the 6-month follow time point. At the earlier time point, counselors felt quitters would be more engaged and appreciative of the Quitline, and perhaps more motivated to encourage others to quit also. Also, the pool of smokers to recruit as peer-navigators would be considerably larger the earlier the recruitment occurred, as many smokers do not complete six-month follow-up calls.

Another solution may be to turn to 21^st^ century technology, replacing the paper referral with text-messaging-based referral, creating not only a simpler process, but a ‘warm handoff’. The result of text-messaging referrals is that the Peer-referrer would not have to remember to carry around to referral forms, they could use their phone. Also, the Peer-referrer would not have to wait until the forms were completed, and the Quitline would not have to wait for the mail to arrive. Thus, enrollment attempts could be swifter.

Another way to engage individuals may be to increase the incentives for peer referrals, especially for those participants in rural areas. Examples could include payments for a certain number of referrals, payment for referrals that register with the Quitline, or additional free NRT. Quitlines are paid by volume, and perhaps outsourcing the workload, supporting the Quitline with additional NRT free, or providing payment to the Quitline for engaged smokers may be feasible incentives for participation.

## Conclusions

By tapping into social networks and having champions of the Quitline process, we hoped to reach a new group of hard-to-reach smokers in these communities. Through our pilot we have provided a unique initial demonstration of the challenges, and propose in future research strategies for streamlining the process and recruiting newly-quit smokers who may be more likely to be engaged in the Quitline.

## Availability of supporting data

The data sets supporting the results of this article are included within the article and its additional files.

## Competing interests

The authors declare that they have no competing interests.

## Authors’ contributions

KD contributed to the research, writing and analysis of the article; JV contributed to the writing and review of this article; BP contributed to the analysis and writing of this article; TH contributed to the design of the research, writing and analysis of the article. All authors read and approved the final manuscript.

## Supplementary Material

Additional file 1 Tobacco Quitline. Click here for file
