# Supplementary material for: Quitters referring smokers: a quitline chain-referral pilot study
Source: BMC Res Notes. 2014 May 5;7:282. doi: 10.1186/1756-0500-7-282 (PMC4108050; doi:10.1186/1756-0500-7-282)
Supplement: Additional file 1 — Tobacco Quitline. [file 1756-0500-7-282-S1.doc]

Additional file

**Tobacco Quitline**

**1-800-Quit-Now**

(1-800-784-8669)

------------------------------------------------------------------------------------------------------------------------------------------

ID Number: _[*Peer Navigator ID Auto-generated from Quitline*]____

Name: _______________________________________________________________________

**Free, Personalized Help. Just for you!**

The Tobacco Quitline if a FREE telephone-based smoking cessation program. A trained professional counselor will call you to provide information to help you succeed in quitting tobacco. The Quitline offers:

• Private counseling and support

• A quit plan made just for you

• Skills to help you succeed in quitting

• Tobacco quit material mailed to you

• Free nicotine replacement therapy for up to 4 weeks

The counselor helps you figure out what works best for you. A counselor doesn’t tell you what to do. You work with a counselor to make changes that fit your life.

Relationship to Peer Navigator (Circle One):

Spouse Friend Relative Other (Please specify): ____________________

Telephone Number: ( __)___________________ May We Leave a Message: 🞏Yes 🞏No

The Tobacco Quitline staff can call me during the following times (Check all that apply):

🞏 7am–10am 🞏10am–1pm 🞏1pm–4pm 🞏4pm–7pm 🞏 7pm–9pm

Signature for Consent: _____________________________________________

I hereby authorize ____[*Peer Navigator Name*]______ to release my contact information to the Tobacco Quitline. This authorization is continuing. I understand that the Tobacco Quitline will contact me to provide information and offer support in quitting tobacco. My participation is voluntary. I understand that any information I provide will be kept confidential.
